# Recreational Centres’ Facilities and Activities to Support Healthy Ageing in Singapore

**DOI:** 10.3390/ijerph16183343

**Published:** 2019-09-10

**Authors:** Elaine Yee-Sing Wong, Andy H. Lee, Anthony P. James, Jonine Jancey

**Affiliations:** 1 School of Public Health, Curtin University, GPO Box U1987, Perth 6845, Australia; 2 Collaboration for Evidence, Research and Impact in Public Health, (CERIPH), School of Public Health, Curtin University, GPO Box U1987, Perth 6845, Australia

**Keywords:** age-friendly, facilities, healthy eating, health behaviour, health promotion, lifestyle, physical activity, recreational centres

## Abstract

Objective: This study examined the physical and social environment (facilities and activities) of Singapore’s Recreational Centres (RCs) and female patrons’ (>50 years) perception of the RC facilities and activities. Materials and Methods: A total of 100 RCs were audited, and 22 face-to-face interviews were undertaken. Results: Physical activity classes were the main activity offered (mean = eight classes per month), with walking (29.8%) and aerobics sessions (17.5%) being the most frequent. Nutrition classes and social activities were offered less often (mean = one class per month). The activities were well received by patrons, offering opportunities to interact while participating in physical activity and nutrition classes. However, the need for staff training, consideration of patron’s abilities and the desire to engage in alternative activities were expressed. Conclusion: Overall, RCs’ facilities and activities were well liked by the patrons but opportunities for improvements were identified. Regular reviews of facilities and activities through consultation with the RC patrons and managers are needed to ensure that the facilities and activities remain relevant and practical to the patrons. This will help to support active lifestyles and healthy eating practices among older adults residing within the community.

## 1. Introduction

Inadequate levels of physical activity (PA) and poor dietary behaviours are key modifiable risk factors for non-communicable diseases (NCDs) [1]. These risk factors contribute to two in five deaths worldwide and 30% of the global NCD burden [2]. Studies worldwide have demonstrated that community settings, such as Recreational Centres (RCs), are promising environments to support PA and dietary behaviours and enable individuals to adopt and sustain a healthy lifestyle [3,4,5,6]. The local environment surrounding the RCs can potentially provide opportunities to support PA (traffic-free and safe walking routes) or inhibit PA (poor lighting and uneven walkways) [7,8,9]. In addition, the environment can also encourage healthy eating (communal fruit and vegetables garden) or inhibit healthy eating (presence of unhealthy foods in vending machines) [10,11]. Recent systematic reviews have reported that adults are more physically active when living in close proximity to recreational destinations such as health services, food outlets, parks and shops [12,13,14], while environments with connected streets, good public transport and ease of pedestrian crossing are positively related to increased PA [4,7,12]. Interventions embedded in the community have also been found to increase social opportunities, strengthen social networks among friends and family members that facilitate healthy eating and active urban living practices [15,16].

Despite the strong evidence that the RC environment (physical and social) may support PA and dietary behaviours [3,4,5,6,17], limited research has been undertaken in the over-50 group in Asian communities [18]. Most studies reporting on the effectiveness of community-based lifestyle programs originated from Europe, Canada, USA and Australia. Based on the findings from these studies, further research is recommended to explore the influences (social contact, environmental opportunities and program enjoyment) for effective lifestyle programs, highlighting the need for research on Asian communities and especially the unique RC setting in Singapore.

As Singaporeans age, they become increasingly sedentary. Results from the National Health Survey 2010 revealed that the majority (63%) of the 50–69 years old participated in no leisure time PA, while also consuming high amounts of low-nutrient, energy-dense meals [19,20]. A high proportion of older Singaporean adults aged over 50 years had pre-obesity (34%), high levels of abdominal fatness (32%) and impaired glucose tolerance (22%), with higher rates occurring among older women [20]. These behavioural and metabolic risk factors increase their risk of NCDs [21]. Cardiovascular diseases, cancer and diabetes account for 64% of the disease burden in Singapore [22], as one-quarter of adults aged over 65 have at least one NCD [23]. The Singapore government has invested significantly in the management of chronic diseases such as cardiovascular diseases (US $8.2 billion) [24] and diabetes (US $0.7 billion) annually [25]. 

Considering the high cost of NCD management, the high prevalence of metabolic risk factors and unhealthy lifestyle practices among older Singaporean adults [26], there is an urgency to examine the physical and social environments to develop effective community-based programs to enhance health behaviours. Multi-component strategies in health programs that include group education, PA classes and dietary counselling sessions have resulted in improvements in physiological outcomes [27,28,29]. Additionally, available evidence indicates that there is some cost-effectiveness in community health programs [30]. Studies undertaken in Western developed countries have reported achieving lower cost per quality adjusted life years (QALYs) through health programs conducted for a longer duration [31]. These programs focused on different NCDs, such as diabetes and cardiovascular diseases, and ranged from US$1413 to US$7301 per QALY gained for adults, depending on the extent, duration and resources of the program [31,32,33,34,35]. Singapore’s unique RCs may provide an opportunity to promote cost-effective lifestyle programs to support health-enhancing behaviours.

Singapore’s RCs are communal spaces built in the 1970s to support a sense of community among residents who had moved out of their rural village into these urban Housing Development Board estates [36]. These RCs were built below the dense high-rise public housing, where more than 80% of Singaporeans now reside [37]. These public communal spaces serve as a venue for residents to undertake activities, interact and foster community cohesiveness [38]. There are 579 RCs located across the five major districts in Singapore (Central (n = 164), North-East (n = 140), South-West (n = 115), North-West (n = 106) and South-East (n = 54)), each with a similar socioeconomic status [39]. These RCs are staffed with managers who collaborate with the Health Promotion Board (HPB) to promote health and wellness activities [40]. The HPB has been tasked with the vision of building “a nation of healthy people” by encouraging health-enhancing behaviours and preventing illness, disability and premature death [41]. The majority of patrons who frequent these RCs are women aged 50 years and over [42,43], providing a local community environment to support positive changes in PA and dietary behaviours for this ‘at-risk’ group [44], yet little evidence has been documented [4,45], especially how the RCs can engage and empower these older Singaporean women in healthy lifestyles. 

The present study provided the first report to address this gap in the literature. It aimed to examine the physical environment (facilities and activities) of the RCs and female patrons’ (>50 years) perception of the RC facilities and activities. The study was underpinned by the social-ecological model and Social Cognitive Theory (SCT) due to the interplay between various health determinants. The model aids in understanding the complexity of health behaviours [46,47], emphasizing the multiple levels of influences (individual, interpersonal, community) that shape health behaviours within the environment [48]. The principles of the model are consistent with SCT concepts, which recognise that people are not only driven by inner forces or shaped by external influences [49], but also by human behaviour, cognition, personal factors and the environment [50]. Both quantitative (assessment of the physical environment) and qualitative (interviews with RC patrons to assess their experiences) data were collected to identify activities and facilities offered by the RCs, and the patrons’ perceptions of these activities and facilities. Such information will assist our understanding of these facilities and their usage, which is important for the development of strategies for cost-effective programs to increase engagement with RCs. 

## 2. Materials and Methods 

### 2.1. Recreational Centre Recruitment

Of the 579 RCs located across the five major Singapore districts (Central, South West, North West, South East and North East), 121 were randomly selected using an online computer random number generator (see Table 1). The RC managers were contacted to explain the purpose and scope of the study. Among them, 100 managers agreed to participate. They were informed of their rights and that the audit would be kept strictly confidential and non-identifiable. A suitable time was then organised for one of the five trained research assistants (qualified nutritionists or certified fitness instructors) to visit the RC, discuss the centre’s profile and undertake the audit. 

The standardised training of the research assistants was conducted by the principal investigator (PI; first author). They received detailed written instructions on the audit tool usage and an assessment manual. The PI further demonstrated use of the audit tool and explained the interview schedule. This was then followed by a feedback session to clarify any issues. Thereafter, research assistants conducted the audits and interviews independently. Random inspections were made to ensure that the audits and interviews were conducted properly. Permission to conduct the audit was sought from the RC managers before proceeding with the systematic observation, which was performed inside the RC premises and the surrounding area and took approximately 90 minutes to complete. Informed consent was obtained from each participant before the face-to-face interview. The research protocol was approved by Curtin University Human Research Ethics Committee (approval number: HRE2016-0366) and registered on the Australian and New Zealand Clinical Trials Registry (trial no: ACTRN12617001022358).

### 2.2. Audit Instrument

The ‘Audit of Physical Activity Resources for Seniors’ (APARS) is designed to objectively assess features of the building and surrounding areas as well as its facilities and activities. It has high inter-rater reliability, with some evidence of construct validity in assessing health-related resources in the environment [51], and has been previously used by our research team [52]. Older adults aged 50–65 years are frequent users of the RCs [42,43]. Therefore, the APARS tool was considered suitable for this assessment. The audit was adapted for the Singaporean context to identify facilities and activities (PA, nutrition and social) provided by the RCs. The audit tool assessed the presence of facilities, i.e., ‘Exterior PA facilities’, ‘Inside PA and nutrition facilities’, ‘aesthetics’, i.e., ‘Exterior environmental features’ as well as ‘Inside social activities’. RC health activities were classified as ‘PA activities’, ‘social activities’ and ‘nutrition activities’. In addition, the profile of each centre was also constructed by gathering data on their characteristics and patrons as provided by the RC managers (see Table 2).

### 2.3. Face-to-Face Interview Recruitment

From the 100 audited RCs, 22 RC patrons (at least four participants from each district) were purposefully selected to participate in the interviews. All participants selected were women aged 50 years and over who were currently attending the RCs and residing within the respective neighbourhood. These participants were approached and consecutively recruited at the RCs. After obtaining their informed consent, interviews of approximately 30 minutes duration were conducted in a quiet private location outside the RC. 

### 2.4. Interview Schedule

An interview schedule was developed (see Appendix A) to explore participants’ perception of facilities and activities offered at the RCs. The interview asked what participants liked about the facilities and activities, what they did not like, and how these could be improved. Interviews were conducted by the trained research assistants in the most suitable language, which included English, Mandarin and other Chinese dialects.

### 2.5. Data Analysis

Descriptive statistics were used to profile the characteristics of the RCs and demographics of the participants, performed using the Statistical Package for Social Science version 25 [53]. Qualitative data from the interviews were translated from Mandarin or other Chinese dialects to English and transcribed within two weeks after interview. An inductive approach was adopted to analyse the data in order to identify emerging themes [54]. Transcribed data were coded by the PI to form common categories, supported by direct quotes from the de-identified participants. Transcripts and qualitative data analysis were managed using the NVivo software version 11 (QSR International Pty Ltd, Melbourne, Australia) [55]. 

## 3. Results

### 3.1. Profile and Audit of RC Facilities and Activities 

The mean age of the RC attendees was 60 years and predominantly female (82%). The average building size of the RCs was 104.62 square metres (approximately 40% the size of a tennis court), and 80% of the RC managers had been involved in HPB initiatives for approximately 14 months. Eighty percent of these RCs received government funding for activities (see Table 2). 

### 3.2. RC Facilities and Activities 

As shown in Table 3, most RCs were located close to parks and gardens (83%), grassy areas (72%), had access to bicycle racks (73%) and coffee corners with benches (58%). Outside PA facilities included exercise/fitness stations (80%), basketball/badminton courts (60%) and bike paths (41%). Facilities inside the RCs included kitchen (36%), fitness space (26%) and nutrition and PA hard copy resources (41%). Nearby amenities included a coffee shop/food court (98%), medical/dental clinic (95%), bus stop and train station (94%), supermarket/wet market (92%), convenient store (92%), gym and community centre (84%), pharmacy (65%) and physiotherapist clinic (15%). Most centres (87%) offered PA classes, fewer offered nutrition classes (60%) and social activities (37%). On average, approximately eight PA classes were held per month, with walking (29.8%) and aerobics sessions (17.5%) being the most frequent activities, whereas only one nutrition class or social activity was offered per month. 

### 3.3. Face-to-Face Interviews

A total of 22 female RC patrons (mean age 65 years) consented to be interviewed. As shown in Table 4, they were predominantly of Chinese descent (96%), had a partner (96%), achieved primary school education (55%) and resided approximately seven minutes walking distance from their RC. The majority of them attended RC programs on a weekly basis (59%).

Feedback from participants was grouped into two main categories: (a) RC facilities and activities (both positive and negative aspects); and (b) Suggestions to improve facilities/activities. Supporting quotes from individual participants (P#) are provided below the identified themes when appropriate. 

### 3.4. RC Facilities and Activities

#### 3.4.1. Positive Aspects

Most participants commented that the RCs were conveniently located and met their needs—providing a place to engage with their neighbours and increase their social interaction opportunities.

*“It is just a stone’s throw from home…. I go to enjoy the facilities …”.*

(P17)

*“In the past, I did not really know my neighbours, did not interact much... But with the availability of the coffee corner (at the RC), I use the facility to interact … to make more friends”.*

(P20)

Participants enjoyed the range of activities and described reasons for their continued participation and attendance at the RCs. These reasons included improved mental health, increased social interaction with friends and family, and reinforcement of a healthy lifestyle.

*“My qigong classes made me stay physically healthy and active”.*

(P12)

*“Stretching classes gave me more time to spend with my husband”.*

(P14)

*“I learn about using healthy cooking tips at home and when I am buying groceries”.*

(P17)

RC managers were nominated by the participants as playing a critical role in motivating their involvement in the activities offered.

*“RC staff are very nice, approachable and friendly. I made friends with them as they frequently contacted and motivated us to attend upcoming events”.*

(P13)

*“RC managers and volunteers do a good job to reach out to the community and benefitted me by increasing my social circle”.*

(P12)

#### 3.4.2. Negative Aspects

Participants indicated that the limited floor area of the RCs made it difficult to accommodate more participants and restricted the facilities provided and the types of activities offered.

*“Yoga classes are limited by the small facility where participants cannot do much with limited space”.*

(P4)

*“Increasing space may cater to more participants, more activities and bigger events. More funding for communal space can accommodate more people for dance activities”.*

(P7)

Participants nominated several reasons that prohibited them from attending RC activities, such as lack of time due to personal commitments, lack of companionship, physical limitations, language barriers and financial difficulties in paying for the activities.

*“I have limited time with family commitments and work occasionally elsewhere”.*

(P17)

*“My schedule clashes due to caring for my grandchildren”.*

(P20)

*“I prefer to speak my own dialects, i.e., Hokkien or Teochew”.*

(P11)

*“Classes are too strenuous for us”.*

(P15)

*“Each RC session costs US$2-$5 …. We would prefer ‘free’ sessions”.*

(P2)

### 3.5. Suggestions to Improve the Facilities/Activities

Participants proposed age-friendly indoor and outdoor safety fixtures (handrails, non-slip floors) and aesthetically pleasing spaces. Increased government funding to upgrade facilities was also seen as important.

*“Facilities should be more user friendly, such as handrail, grab bar with slip resistant toilets”*.

(P4)

*“RCs can be beautified with more plants, flowers, herbs and spices”.*

(P14)

*“Government can enhance aesthetics of the RC, i.e., waterfall area, mini bonsai gardens and bear the cost of grab bars and slip resistant flooring”.*

(P18)

Additional RC facilities suggested by the participants were diverse. These included a library, karaoke room, dance room, kitchen studio, reading corner and wellness centre.

*“Some folks are very good at singing; a karaoke room can display their talent and boost their self-esteem”.*

(P3)

*“A dance room could keep us fit. By expanding the kitchen studio, more people can participate in cooking demonstrations”.*

(P7)

Several participants indicated that activities should be social, of interest to them, and free of charge, with incentives seen as attractive motivators for RC participation. Preferred incentives included supermarket vouchers, discounts, reward cards, healthy food product samples, sports towels and Fitbits.

*“Low cost educational tours are very enjoyable...we explored new culture, food and sights which foster relationships when travelling as a group”.*

(P2)

*“Sponsorship products such as nuts, sesame oil, wholegrain products can encourage healthy eating, or fitness gears to increase PA, such as a Fitbit”.*

(P4)

*“Government can provide RCs with discount cards, reward cards to use at healthier dining outlets”.*

(P15)

Approaches to increase engagement with RC activities included matching of activities to participant’s interests and abilities, involving family members, training multi-ethnic older volunteers to run activities, and linking external parties to share expertise.

*“I love art and craft in making pretty and beautiful things using my hands”.*

(P11)

*“Retro or traditional Chinese music is good for dancing but at a slower pace for inactive people like me”.*

(P15)

*“Ensure most activities have focus on family members… cooking competition can involve the children, parents and grandparents together”.*

(P7)

*“Reach out to minority groups…. An older leader from different races could lead the fitness class or cooking session”.*

(P19)

*“Youth Executive Committee from community centres could be involved in planning of activities to inject more ideas”.*

(P7)

Some participants expressed the need for the additional training of RC managers and more staffing in order to provide activities suited to their interests. Conversely, they also acknowledged that the RC staff do display a genuine interest in promoting healthy ageing initiatives.

*“RC staff should be trained to show initiative, and enthusiasm in caring for the older people”.*

(P4)

*“RC staff could train participants on gardening as plants can be therapeutic for the mind and help to beautify the RCs”.*

(P14)

*“There should be annual seminars for RC staff to share best practices, innovative and successful initiatives in building a more caring community”.*

(P4)

*“We should reward RC staff who drive participants to attend wellness activities, such as health promoter of the month or best RC district manager in health advocacy”.*

(P8)

## 4. Discussion

Despite the promotion of RCs as a recreational venue for older adults and the high demand for such community facilities to support healthy active aging in Singapore [45], there remains limited published information on the facilities and activities offered by RCs and their patrons’ perception of these services [40]. With the exception of a small-scaled study of RC resources [45], the present study represents the first comprehensive report on this important topic. 

There were several issues raised by the participants regarding the RC facilities. Firstly, the average floor space of the RCs was less than half the size of a tennis court, thus restricting the type of PA activities and facilities that could be offered. Space is limited in Singapore, which presents a challenging problem for RC managers to develop creative ways to manage the limited space available while servicing patrons. One solution may be to stagger activities for more patrons to increase access. The concern about limited space in RC indicates the necessity for government support to upgrade and expand RCs, to ensure the continuation of RC activities to meet the social and health needs of Singapore’s ageing population. Eighty percent of the RC managers reported receiving government funding in the last 12 months, but the amount of such funding might not be adequate to upgrade facilities.

The audit revealed more opportunities to engage in PA within the RC environment than nutrition and social activities. The small-scale piloted study, undertaken in seven residential zones within one Singapore district (West region), similarly reported that PA was the main activity offered within RCs and their tendency to prioritise PA [45].

The importance of active lifestyle-enabling features for healthy ageing in cities is well recognised [56]. Examples of such features identified in the present study were hazard-free walkways with adequate lighting, outdoor facilities (park, fitness station, sports court, and bicycle rack) and nearby amenities (eateries, medical clinics, and efficient transport system). These environmental features will enable more PA opportunities, as evidenced in a cross-sectional study of 14 cities which showed increased PA in densely populated neighbourhoods with walkable interconnected streets, close amenities and parks [7]. In addition to these PA enhancing features, the patrons also provided suggestions to support their PA levels, such as “*attractive wall murals with ‘get active’ messages*”, “*walking initiatives at nearby nature trails*” and the *“enhancement of aesthetics of the RCs through installation of a waterfall area or mini bonsai gardens”.* These suggestions should be considered by the RC management. 

The focus on PA is reasonable and acceptable, especially when considering that 61% of Singaporean adults are not meeting the PA guidelines of more than 150 minutes of moderate activity each week for health benefits [20], along with the positive health benefits associated with being physically active [57]. Activities such as walking are inexpensive, requiring no special equipment or skills and, when conducted in groups, can enhance social connectedness [58]. However, the promotion of healthy eating is equally important, since many Singaporean adults aged over 50 years have exceeded the recommended dietary allowance for energy, total fat, saturated fat and carbohydrates [19]. Our study found the RCs provided limited nutrition-related facilities and activities both inside (kitchen (36%) and kitchen appliances (46%)) and outside (vegetable/fruit/spice garden (36%), coffee benches (58%) and vending machines (33%) stocked with healthy snacks). To support healthy dietary behaviours, the social and physical features of the RC could be altered by environmental cues [10,11]. For example, increasing the availability of healthier products in the vending machines would stimulate healthy snacking. RC managers could improve the aesthetic of the coffee benches and the kitchen to increase social interaction, provide healthy culinary courses for patrons, and develop community gardens. Community gardens, for instance, have many benefits that include developing social networks and friendships, increasing a sense of happiness and reducing stress through social interaction, improving access to fresh and nutritious vegetables [59], as well as replacing fast food consumption by fruit and vegetables [60]. A global meta-analysis confirmed the beneficial effects of gardening on mental wellness (depression, anxiety and life satisfaction), health outcomes (body mass index and quality of life) and general health [61]. The installation of community gardens is possible since 72% of the RCs were observed to occupy more than one grassy area (> 6 m × 6 m) outside their premises.

Based on the principles of the socio-ecological model and SCT concepts, the interaction of the interpersonal, physical environment and socio-cultural factors can either inhibit or facilitate engagement in health behaviours [46,62]. Several factors facilitated the engagement of patrons to utilise the RC, as reflected by their opinion: “*It is just a stone’s throw from home”, “enjoy the facilities”, “use the facility to interact”, “make more friends”, and “stretching classes gave me more time to spend with my husband*”. On the other hand, factors that hindered their RC involvement should be addressed: *“prefer to speak my own Chinese dialect”* and “*classes are too strenuous for us*”. They also suggested a range of strategies to optimise engagement with RCs, such as increasing staff and volunteer training, recognition of staff commitment, involving families, provision of healthy incentives and considering patron’s interests and abilities. Participants suggested alternative activities, such as “*making pretty and beautiful things with my hands”, “dance of slower pace to cater for the inactive participant*”, and *“gardening as plants are therapeutic and help to beautify the RCs”*. Age-friendly facilities such as the installation of handrails, non-slip floors, kitchen studio, dance and karaoke rooms were also proposed, which are worthy of consideration for future program planning. Moreover, the patrons nominated RC managers as playing a vital role in promoting health. A diverse range of adaptable facilities and customised activities should be provided at flexible times to keep them physically fit and socially active. Early involvement, consultation and engagement between managers and patrons should be encouraged to modify or refine facilities and activities, in order to motivate PA and dietary behaviour change. They further acknowledged the need for RC managers to be educated through annual seminars and be rewarded for their contribution. These findings can be used to better inform government to suit the RC patrons’ changing physical abilities, demands and interests.

Future research should examine the impact of the RC activities and facilities on offer and their cost effectiveness in terms of reducing NCDs. Researchers could collaborate and innovate health promoting RC environments that are conducive to healthy aging. Comparisons between and within various RCs could be conducted to inform ways to manage resources more efficiently and promote best practice strategies and policies that reinforce and accelerate health-enhancing behaviours.

## 5. Conclusions

The capacity for the physical and social environment to positively influence PA and dietary habits has been well documented. This study revealed that RC facilities and activities primarily placed a greater emphasis on PA and less on healthy eating practices. The positive and negative aspects of RCs nominated by patrons should be reviewed by RC managers, along with their suggestions to improve the activities and facilities when promoting health behaviours. In particular, the provision of aesthetically pleasing RC environments, monetary incentives and healthy refreshments were highly recommended by RC patrons to motivate them to stay active and eat well. RCs serve as desirable community hubs to enhance healthy behaviours through appropriate activities and social connectedness. Collaboration between managers and patrons is paramount in making the RC facilities and activities relevant and supportive of active participation in healthy lifestyles. RC patrons have reported a strong interest in programs that focus on building social ties with their families and peers, while regarding RC managers as positive role models in encouraging them to sustain behavioural changes. The development of policy to regularly assess and modify the RC environment could positively instigate PA and healthy eating practices to address the high rates of chronic diseases in Singapore.

## 6. Limitations

The present study represents the most comprehensive report on RCs in Singapore. The interviews were undertaken with female patrons because they constituted the predominant users of RCs (82%). It would be of interest to find ways on how to attract more men to RCs. The qualitative analyses were based on 22 interviews. Future surveys should be undertaken on a large number of participants across RCs to confirm our findings. In addition, once recorded, the interviews were translated from Mandarin and Chinese dialects into English, which might induce minor impacts on our interpretations. Since the study focused on specific types of services in a specific socio-geographical context, the generalisability of the findings is somewhat limited. Finally, future research should consider using cluster randomisation (i.e. same percentage of RCs in each area) to ensure equal representation of RCs.

## Figures and Tables

**Table 1:** Samples of Recreational Centres (RCs) from five Singapore districts.

District	No. of RCs in District	No. of RCs Audited
Central	164	20 (12%)
North East	140	26 (19%)
South West	115	24 (21%)
North West	106	17 (16%)
South East	54	13 (24%)
TOTAL	579	100 (17%)

**Table 2:** Characteristics of Recreational Centres (n = 100).

Patrons	
Age: mean (SD), years	60 (10.3)
Female (%)	82%
No. of patrons per class: mean (SD)	30 (19)
*Recreational Centres*	
Building area: mean (SD), m^2^	104.62 (1.92)
Involved in HPB initiatives (%)	80%
Duration of HPB involvement: mean (SD), months	14 (17.1)
Government funding for patron activities (%)	80%

HPB—Health Promotion Board; SD—standard deviation.

**Table 3:** Facilities and activities at Recreational Centres (n = 100).

**Exterior Environmental Features**	
Presence of parks and gardens (within 400 m)	83%
Bicycle racks	73%
> 1 grassy area (> 6 m × 6 m)	72%
Coffee corner with benches	58%
Vegetable/fruit/spice garden	36%
Vending machines with healthy foods/drinks	33%
No obstruction on path to centre	97%
Adequate footpaths to centre	94%
≥ 1 exterior light outside centre	80%
**Exterior Physical Activity Facilities**	
Exercise/fitness stations	80%
Basketball/badminton courts	60%
Bike paths	41%
**Inside Nutrition and Physical Activity Facilities**	
Washing basin for food preparation	80%
Utensil/Stove/Wok/Induction cooker/Oven	46%
Health booklets or recipe handouts (PA and nutrition)	41%
Kitchen	36%
Fitness space	26%
Weights/resistance equipment	14%
**Inside Social Facilities**	
Open social lounge or living room area with television	79%
Projector	71%
Board and card games	55%
Dining room	35%
Library	27%
Interactive video games	8%
**Nearby Amenities (< 400 m)**	
Coffee shop/Food court	98%
Medical/dental clinic	95%
Bus stop and train station	94%
Supermarket/Wet market	92%
Convenient store	92%
Gym/Community centre	84%
Pharmacy	65%
Physiotherapist clinic	15%
**Health Activities**	
PA classes: mean (SD) per month	7.87(8)
No. of RCs offering PA classes (%)	87%
Walking	29.8%
Aerobics	17.5%
Qigong	16.2%
Others (ball games, flexibility, martial arts, piloxing and yoga)	7.5%
Dance	7.0%
Tai chi	7.0%
Resistance training (resistance band)	5.0%
Nutrition classes: mean (SD) per month	1 (1.15)
No. of RCs offering nutrition classes (%)	60%
Cooking demonstrations	75.8%
Nutrition talks	24.2%
Social activities: mean (SD) per month	1 (3.19)
No. of RCs offering social activities (%)	37%
Mahjong	38.0%
Rummy O	33.3%
Bingo	28.6%

SD—standard deviation; PA—physical activity; Qigong—a holistic system of coordinated body posture and movement, breathing, and meditation; Mahjong—a tile-based game developed in China. It is commonly played by four players and is a game of skill, strategy and calculation; Piloxing—a system of exercise combining elements of Pilates and boxing; Rummy O—a tile-based game for two to four players based on matching tiles of the same rank or sequence and same suit; Tai chi—an ancient Chinese discipline of meditative movements, practiced as a system of exercises.

**Table 4:** Profile of interviewed patrons (n = 22).

Age: mean (SD), years	65 (8.8)
**Ethnicity, n (%)**	
Chinese	21(95.5%)
Malay	1 (4.5%)
**Marital status, n (%)**	
With partner	21(95.5%)
Without partner	1 (4.5%)
**Education level, n (%)**	
Primary school	12(54.5%)
Secondary school	8 (36.4%)
University	2 (9.1%)
Distance from residence to centre: mean (SD), walking mins	7.39 (6)
**Frequency of attending Recreational Centre, n (%)**	
Daily	6 (27.3%)
Weekly	13 (59.1%)
Monthly	3 (13.6%)

SD—standard deviation.

## Abbreviations

APARS	Audit of Physical Activity Resources for Seniors
HPB	Health Promotion Board
QALY	Quality-adjusted life-years
NCD	Non-communicable diseases
PA	Physical activity
PI	Principal Investigator
RC	Recreational Centre
SCT	Social Cognitive Theory
SD	Standard Deviation

## Appendix A. Interview Schedule

- What do you like about the facility/facilities?
- What do you like about the activity /activities?
- What activity/activities do you participate in?
- What Recreational Centre facilities would you will like to see more/less of? Why?
- What activities would you like to participate in more/less of? Why?
- Has the Recreational Centre facilities and activities benefited you?
- What do you think motivates you to use the Recreational Centre facilities?
- What do you think motivates you to participate in the activities?
- What do you think would make you more likely to use the Recreational facilities and the activities?
- What do you think Recreational Centre managers and volunteers could do to improve the current facilities and activities in the community?
- What suggestions do you have on how government or other agencies could help to improve the Recreational Centres to support the health status of residents?
